# Assessing Liver Function in Liver Tumors Patients: The Performance of T1 Mapping and Residual Liver Volume on Gd-EOBDTPA-Enhanced MRI

**DOI:** 10.3389/fmed.2020.00215

**Published:** 2020-05-28

**Authors:** Ting Duan, Hanyu Jiang, Chunchao Xia, Jie Chen, Likunn Cao, Zheng Ye, Yi Wei, Bin Song, Jeong Min Lee

**Affiliations:** ^1^Department of Radiology, West China Hospital, Sichuan University, Chengdu, China; ^2^Department of Radiology, Peking Union Medical University Hospital, Peking, China; ^3^Department of Radiology, College of Medicine, Seoul National University, Seoul, South Korea

**Keywords:** Gd-EOB-DTPA, T1 relaxation time, residual liver volume, magnetic resonance imaging, liver function

## Abstract

**Purpose:** To assess the performance of T1 mapping and residual liver volume (RLV) on Gd-EOBDTPA-enhanced MRI in pretreatment estimation of liver function in patients with liver tumors. Indocyanine green retention rate at 15 min (ICG R-15) was used as a reference standard.

**Methods:** Ethical approval from the institutional review board and informed consent were obtained for this prospective study. We enrolled 155 patients with liver tumors who underwent pretreatment Gd-EOB-DTPA-enhanced MRI. T1 relaxation time before (T1-pre), 20 min after (T1-post) Gd-EOB-DTPA injection and RLV were measured. The absolute reduction (ΔT1) and reduction rate (ΔT1%) of T1 relaxation time, volume-assisted ΔT1 (ΔT1^*^RLV) and volume-assisted ΔT1% (ΔT1%^*^RLV) were calculated accordingly. The correlation of MR parameters with ICG R-15 was determined using Spearman's rank correlation analysis. Patients were classified into the normal liver function (NLF) group if their ICG R-15 levels were <10% or otherwise into the abnormal liver function (ALF) group. Receiver operating characteristic (ROC) analysis was conducted to evaluate the performances of the MR parameters in predicting ALF.

**Results:** T1-post (*r* = 0.472, *P* < 0.001), ΔT1 (*r* = −0.355, *P* = 0.011), ΔT1% (*r* = −0.482, *P* < 0.001), RLV (*r* = −0.336, *P* < 0.001), volume-assisted ΔT1 (*r* = −0.458, *P* < 0.001) and volume-assisted ΔT1% (*r* = −0.522, *P* < 0.001) showed weak to moderate correlation with ICG R-15. The area under the ROC curves (AUROC) of volume-assisted ΔT1 in predicting ALF was 0.777, which was significantly higher than the other parameters (*P* < 0.05 for all).

**Conclusions:** Combined T1 mapping and RLV on Gd-EOB-DTPA-enhanced MRI can help assess liver function with good diagnostic accuracy in patients with liver tumors before treatment.

## Introduction

Despite technical advances in locoregional treatments including radiofrequency ablation (RFA) and transarterial chemoembolization (TACE), liver resection (LR) remains the most effective treatment for liver tumors at present (1). Recent advances in hepatic surgery and perioperative care have substantially improved patient outcomes after LR (2). However, with morbidity ranging from 10 to 40%, postoperative liver failure is still one of the most important factors leading to poor prognosis (3). Liver failure can also occur after RFA and TACE, particularly in patients with large tumors or limited hepatic function reservoirs (4). A previous study has shown that a high residual to total liver volume ratio (RLV/TLV) was required for patients with an impaired liver function to tolerate resection (5). Therefore, accurate preoperative evaluation of liver function plays a crucial role in clinical decision making to avoid liver failure after LR. In order to evaluate liver function, comprehensive scoring systems including Child-Pugh class and the model of end-stage liver disease (MELD) scores have been developed (6, 7). In addition, indocyanine green (ICG), a water-soluble anionic compound that is selectively taken up by hepatocytes and excreted unchanged into the bile, is widely evaluated as a tool for liver function assessment in surgical candidates (8). Several studies have reported that ICG retention rate at 15 min (ICG R-15) is effective for the pretreatment evaluation of the hepatic functional reserve and can serve as a significant predictor of postoperative liver failure and mortality (9). However, these clinical scores and ICG test may provide information regarding global liver function rather than regional deterioration of liver function.

As a functional sequence, magnetic resonance imaging (MRI) T1 mapping sequence with hepatobiliary contrast agent gadolinium ethoxybenzyl diethylenetriamine pentaacetic acid (gadoxetic acid or Gd-EOB-DTPA) is an alternative noninvasive imaging approach to measure liver function in both globally and regionally. Gd-EOB-DTPA-enhanced MRI permits the assessment of vasculature and hepatocyte function in a single examination and has led to greatly improved detection and characterization of liver tumors (10, 11). Gd-EOB-DTPA has been reported to have a T1-shortening effect; thus, measuring T1 relaxation time of the liver parenchyma before and after Gd-EOB-DTPA injection enables quantitative evaluation of the uptake of the Gd-EOB-DTPA and liver function (12). Additionally, residual liver volume (RLV), which contains the anatomic information, is another parameter that is significantly correlated with hepatic functional reserve in patients treated with LR and TACE (13, 14).

However, although several previous studies have confirmed the performance of T1 mapping or RLV in assessing liver function, just a few have combined these parameters (15–17). Therefore, the purpose of this study is to evaluate the performance of T1 mapping and residual liver volume (RLV) on Gd-EOB-DTPA-enhanced MRI, using ICG R-15 as a reference standard, in the pretreatment estimation of liver function in patients with liver tumors.

## Materials and Methods

### Patients

Ethical approval by the institutional review board by West China Hospital and informed consent were obtained for this prospective study. From November 2015 to December 2017, consecutive patients who were clinically suspected to have liver tumors based on their clinical history, physical examination and ultrasound results from our center were enrolled. Patients (1) with any previous surgery of the liver or biliary system, TACE, RFA, percutaneous ethanol injections, or chemotherapy within 2 months before MRI examination; (2) with any contraindication of MRI (e.g., pacemaker, claustrophobia, allergy to contrast media, or renal dysfunction); (3) with poor MR image quality for reliable qualitative or quantitative assessment; (4) or without ICG test results were excluded.

### MRI Protocols

All examinations were performed on a MAGNETOM Skyra 3T MR scanner (Siemens Healthcare, Erlangen, Germany) with an 18-channel body array coil. Patients were scanned in the supine position after they had fasted for 6–8 h. Routine MR sequences included breath-hold T2-weighted imaging, heavily T2-weighted imaging, diffusion-weighted imaging (b values: 0, 50, 500, 800, 1,000, and 1,200), and T1-weighted dual-echo gradient imaging. For dynamic imaging, a standard dose of Gd-EOB-DTPA (0.025 mmol/kg, Primovist® Bayer-Schering Pharma AG, Berlin, Germany) was intravenously injected at a rate of 2 mL/s, immediately followed by a 30-mL saline flush. Images in arterial, portal venous, transitional, and hepatobiliary phases (HBP) were obtained by using a T1-weighted three-dimensional (3D) gradient-echo sequence (volume interpolated breath-hold examination, VIBE) with fat suppression. HBP 3D VIBE sequence was used to obtain anatomic images.

Functional images were aquired by T1 mapping sequences (Look-Locker), which were performed before and 20 min after the injection of Gd-EOB-DTPA, with a 180° inversion recovery (IR) at the beginning, and followed by continuous fast low angle shot (FLASH) acquisitions. Other imaging parameters were set as follows: repetition time (TR), 3.00 milliseconds (ms); echo time (TE), 1.32 ms; flip angle, 8°; field of view (FOV), 380 × 300 mm2; matrix, 192 × 153; and slice thickness, 4.0 mm with an interpolated 0.8-mm section thickness. A parallel imaging technique with an acceleration factor of 2 (iPAT = 2) was applied using generalized autocalibrating partially parallel acquisition (GAPPA).

### T1 Mapping Image Analysis

**Figure 1:**
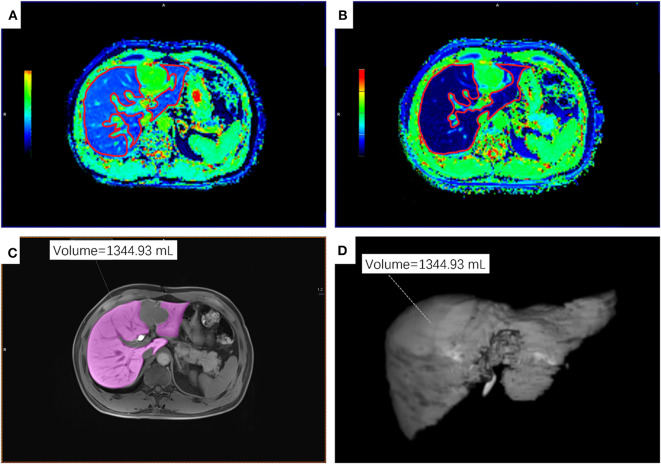
An example of ROIs on T1 maps, T1-weighted VIBE sequence and 3D volumetric images of the residual liver parenchyma. **(A,B)** ROIs on the T1-mapping before **(A)** and 20 min after **(B)** the administration of Gd-EOB-DTPA. **(C)** ROI on T1-weighted VIBE sequence. **(D)** 3D volumetric image. ROI, regions of interest.

Quantitative T1 relaxation time maps were derived automatically on a voxel-by-voxel basis. The MR data sets were transferred to a Singo Via 127 workstation (Siemens Healthcare, Erlangen, Germany) to measure the T1 relaxation time of the liver parenchyma within the operator-defined regions of interest (ROIs). The ROIs on T1-pre and T1-post images covering the whole liver were manually placed on the same section, carefully excluding 5 mm of the liver border to avoid partial volume effects, any visible focal lesions, and all major vessels (Figures 1A,B). The ROIs were drawn independently by 2 radiologists (Ting Duan and Hanyu Jiang) with 4 and 5 years of experience in liver MR imaging, respectively, who were blinded to all clinical information. T1 relaxation times were measured as T1-pre (before administration of Gd-EOB-DTPA) and T1-post (20 min after contrast agent injection). The averaged RLV, T1-pre and T1-post between two radiologists were used for further analyses. The absolute reduction (ΔT1) in and reduction rate (ΔT1%) of T1 relaxation time were calculated as follows:

$$\begin{array}{lll} \Delta T1 & = & T1pre-T1post\\ \Delta T1\% & = & 100\%\times \left(T1\text{pre}-T1\text{post}\right)/T1\text{pre} \end{array}$$

### Anatomic and Volumetric Analysis

The same two radiologists as described above delineated the non-neoplastic residual liver parenchyma on each section of HBP 3D VIBE sequence to retrieve the RLV using the application Tissue Segmentation at the same workstation. The freehand outlines were drawn to avoid any visible focal lesions and major vessels (Figures 1C,D).

Volume-assisted ΔT1 were defined as ΔT1^*^RLV and volume-assisted ΔT1% as ΔT1%^*^RLV.

### ICG Test

ICG R-15 was determined to calculate liver function 24–48 h prior to or after MRI examination to eliminate any possible confounding interaction with MR relaxometry within a reasonable timeframe. According to the Makkuchi Criteria (18), patients were classified into the normal liver function (NLF) group if their ICG R-15 levels were <10% or otherwise into the abnormal liver function (ALF) group.

### Biochemical Blood Parameters and MELD and Child-Pugh Scores

Preoperative biochemical blood parameters, including serum alanine aminotransferase (ALT) level, aspartate aminotransferase (AST) level, bilirubin level, creatinine level, and the international normalized ratio (INR) for prothrombin time were measured and recorded. MELD scores were calculated according to the following formula (19): MELD = 3.78^*^ln serum bilirubin (mg/dL) +11.2^*^ln INR+9.57^*^ln serum creatinine (mg/dL)+6.43. Child-Pugh scores were evaluated according to the composite score of five variables, namely, bilirubin, albumin, ascites, encephalopathy, and the INR (20).

### Statistical Analysis

All statistical analyses were performed using the Statistical Package for the Social Sciences (SPSS, version 22.0, SPSS Inc, Chicago, IL). The intraclass correlation coefficient (ICC) was calculated to assess the inter-observer consistency of all MR parameters. The correlation coefficients between ICG R-15 and all averaged MR parameters (RLV, T1-pre, T1-post, ΔT1, ΔT1%, volume-assisted ΔT1 and volume-assisted ΔT1%) were assessed using Spearman's rank-order correlation test in the whole cohort and compared with Student's *t*-tests in NLF and ALF group. The MR parameters of the NLF group and the ALF group were compared using either Student's *t*-tests (if normally distributed) or the Mann–Whitney *U* test (if not normally distributed). Receiver operating characteristic (ROC) analysis was conducted to evaluate the performance of the MR parameters in predicting ALF. Sensitivities and specificities with corresponding 95% confidence intervals (CIs) were derived, and the areas under the ROC curve (AUROCs) were calculated afterward. Differences in diagnostic performance were analyzed by comparing the AUROCs according to the method described by DeLong et al. (21). *P* < 0.05 were regarded to indicate a statistically significant difference.

### Standard Protocol Approvals, Registrations, and Patient Consents

This study was approved by the Blinded Ethics Committee and conformed to the principles of the Declaration of Helsinki. Written informed consent was obtained from all participants.

## Results

### Patient Characteristics

**Figure 2:**
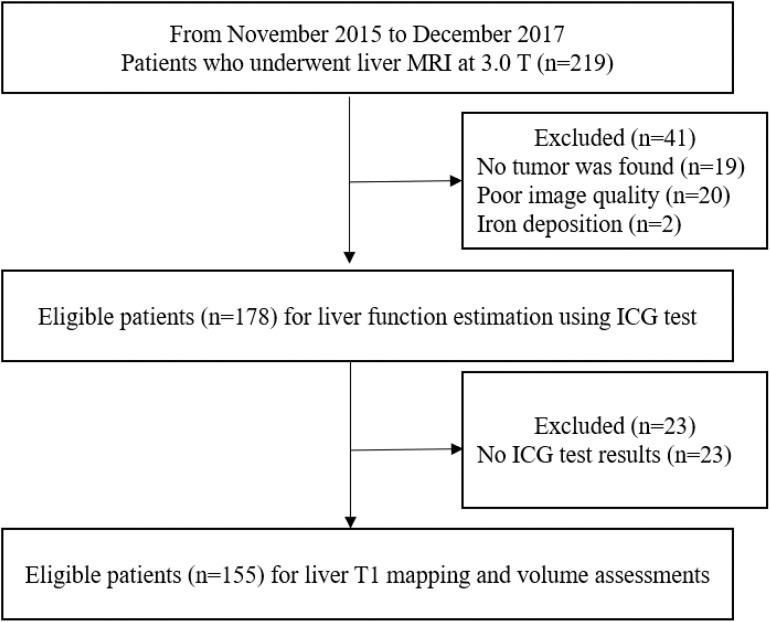
Flow diagram of the study population.

In total, 155 consecutive patients (mean age: 50.21 ± 10.64 years; range: 14–83 years), including 130 men (mean age: 50.31 ± 10.26 years; range: 26–83 years) and 25 women (mean age: 49.46 ± 12.67 years; range: 14–65 years), were recruited. The study flow diagram is shown in Figure 2. Among all patients, 115 were classified into the NLF group and 40 in the ALF group based on ICG R-15 results. AST levels, ALT levels, INR, creatinine levels and MELD scores of the ALF group were significantly higher than those of the NLF group (*P* < 0.05 for all). The demographic information and baseline laboratory findings of the study population in both groups are presented in Table 1.

**Table 1:** Demographics and baseline laboratory data.

	**All patients**	**NLF group (*n* = 115)**	**ALF group (*n* = 40)**	***P***
Age, y (mean ± SD)	50.21 ± 10.64	48.37 ± 10.28	55.53 ± 9.97	0.964
Male/female, n	130/25	98/17	32/8	0.779
Height (cm)	165.40 ± 6.44	165.50 ± 6.59	165.11 ± 6.05	0.399
Weight (kg)	64.44 ± 9.67	64.73 ± 9.88	63.61 ± 9.19	0.460
ICG R-15	7.83 ± 6.26	5.05 ± 2.49	15.80 ± 6.97	<0.001*
**Baseline levels (mean** **±** **SD)**				
Aspartate aminotransferase (IU/L)	42.93 ± 44.07	45.52 ± 43.65	54.85 ± 45.08	0.004*
Alanine aminotransferase (IU/L)	45.53 ± 38.91	43.10 ± 39.01	52.50+38.24	0.028*
Total bilirubin (mg/dL)	16.10 ± 7.72	0.91 ± 0.43	1.03 ± 0.49	0.105
INR	1.03 ± 0.08	1.01 ± 0.08	1.06 ± 0.09	0.003*
Creatinine (mg/dL)	0.75 ± 0.17	0.79 ± 0.17	0.72 ± 0.16	0.002*
prothrombin time (s)	12.17 ± 1.00	12.05 ± 0.95	12.51 ± 1.08	0.506
Albumin (μmol/L)	42.77 ± 5.19	43.96 ± 4.92	39.37 ± 4.41	0.845
Tumor size (cm, mean ± SD)	5.66 ± 3.23	5.82 ± 3.20	5.21 ± 3.31	0.119
MELD score (n)				0.024*
6~7 (*n*)	76	61	15	
7~8 (*n*)	46	34	12	
8~9 (*n*)	17	10	7	
9~10 (*n*)	8	6	2	
10~11(*n*)	5	3	2	
≥11 (*n*)	3	1	2	
Child-Pugh score (n, A/B/C)	152/3/0	115/0/0	37/3/0	0.304

*NLF, normal liver function; ALF, abnormal liver function; AFP, alpha-fetoprotein; ND, not done; SD, standard deviation; ICG R-15, indocyanine green retention rate at 15 min; INR, international normalized ratio; MELD score, model of end-stage liver disease score; TBL, Total bilirubin; AST, Aspartate aminotransferase; ALT, Alanine aminotransferase*.

* *The difference is statistically significant*.

### Inter-observer Consistency

The inter-observer agreement was very good for RLV (ICC = 0.916, 95% CI = 0.855–0.949), T1-pre (ICC = 0.914, 95% CI = 0.852–0.946), and T1-post (ICC = 0.951, 95% CI = 0.909–0.972).

### Correlation Between ICG R-15, Biochemical Blood Parameters, and MR Parameters

A significant negative correlation was observed between ICG R-15 and RLV (rho = −0.336, *P* < 0.001). For T1 relaxation time measurements, T1-post (rho = 0.472, *P* < 0.001), ΔT1 (rho = −0.355, *P* < 0.001) and ΔT1% (rho = −0.482 < 0.001) were significantly correlated with ICG R-15. No significant correlation was found between ICG R-15 and T1-pre (*P* = 0.155). For the volume-assisted parameters, volume-assisted ΔT1 (rho = −0.458, *P* < 0.001) and volume-assisted ΔT1% (rho = −0.522, *P* < 0.001) were negatively correlated with ICG R-15. volume-assisted ΔT1% demonstrated stronger correlations with ICG R-15 than did ΔT1 or RLV (*P* < 0.05 for both). The correlation between ICG R-15, biochemical blood parameters and MR parameters were shown in Table 2.

**Table 2:** Correlation between ICG R-15, biochemical blood parameters and MR parameters.

	**RLV (mL)**	***P***	**T1-pre (ms)**	***P***	**T1-post (ms)**	***P***	**ΔT1 (ms)**	***P***	**ΔT1% (%)**	***P***	**Volume-assisted ΔT1 (ms.mL)**	***P***	**Volume-assisted ΔT1% (mL)**	***P***
ICG R-15	−0.336	<0.001*	0.058	0.475	0.472	<0.001*	−0.355	<0.001*	−0.482	<0.001*	−0.458	<0.001*	−0.522	<0.001*
INR	−0.129	0.112	0.286	<0.001*	0.219	0.006*	0.044	0.591	−0.141	0.082	−0.06	0.458	−0.181	0.025*
TBL	−0.066	0.418	0.028	0.731	0.117	0.147	−0.096	0.236	−0.117	0.147	−0.089	0.273	−0.116	0.149
Creatinine	0.175	0.030*	−0.129	0.110	−0.333	<0.001*	0.174	0.031*	0.311	<0.001*	0.220	0.006*	0.301	<0.001*
ALT	−0.014	0.860	0.026	0.749	−0.041	0.612	0.041	0.609	0.032*	0.696	−0.024*	0.771	−0.021	0.792
AST	−0.158	0.050	0.185	0.021*	0.180	0.025*	0.037	0.646	−0.137	0.088	−0.108	0.183	−0.201	0.012*
Albumin	0.331	<0.001*	−0.222	<0.001*	−0.368	<0.001*	0.131	<0.001*	−0.320	<0.001*	−0.303	<0.001*	−0.431	<0.001*

*NLF, normal liver function; ALF, abnormal liver function; AFP, alpha-fetoprotein; ND, not done; SD, standard deviation; ICG R-15, indocyanine green retention rate at 15 min; INR, international normalized ratio*.

* *The difference is statistically significant*.

### Comparisons Between MR Measurements in Patients in Different Liver Function Groups

**Figure 3:**
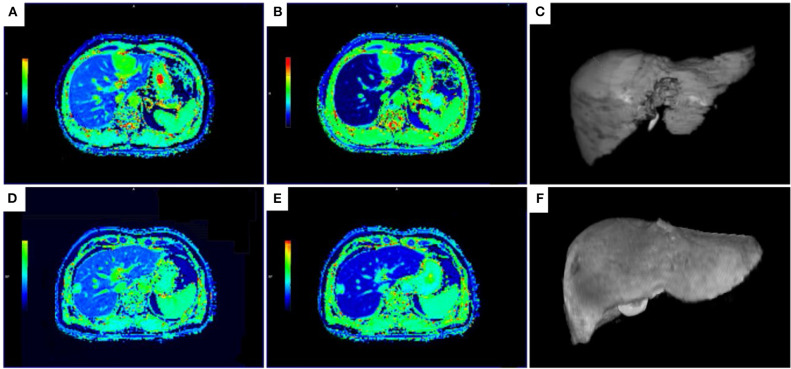
T1 maps and 3D volumetric images of the liver parenchyma for patient with NLF **(A–C)** and ALF **(D,E). (A–C)** The T1-mapping before **(A)** and 20 min after the administration of Gd-EOB-DTPA **(B)** and 3D volumetric image **(C)** in a 47-year-old man with NLF (T1-pre = 694.01 ms, T1-post = 158.08 ms, RLV = 1344.93 mL, ΔT1 = 535.96 ms, ΔT1% = 0.77, volume-assisted ΔT1 = 720828.7 ms.mL, volume-assisted ΔT1% = 1038.60 mL, ICG R-15 = 3.5%). **(D,E)** The T1-mapping before **(D)** and 20 min after the administration of Gd-EOB-DTPA **(E)** and 3D volumetric image **(F)** in a 55-year-old man with ALF (T1-pre = 668.68 ms, T1-post = 328.67 ms, RLV = 915.30 mL, ΔT1 = 340.01 ms, ΔT1% = 0.51, volume-assisted ΔT1 = 311211.2 ms.mL, volume-assisted ΔT1% = 465.41 mL, ICG R-15 = 15.3%). T1-pre, T1 relaxation time before Gd-EOB-DTPA injection; T1-post, T1 relaxation time 20 min after Gd-EOB-DTPA injection; RLV, residual liver volume; ΔT1, absolute reduction of T1 relaxation time; ΔT1%, reduction rate of T1 relaxation time; volume-assisted ΔT1, ΔT1*RLV; volume-assisted ΔT1%, ΔT1%*RLV; NLF, normal liver function; ALF, abnormal liver function.

The RLV (*P* < 0.001), Δ (*P* < 0.05), Δ% (*P* < 0.001), volume-assisted ΔT1 (*P* < 0.001) and volume-assisted ΔT1% (*P* < 0.001) in the NLF group were significantly higher than those in the ALF group, while T1-post in the NLF group was lower than that in the ALF group (*P* < 0.001) (Figure 3). Additionally, T1-pre in the ALF group was higher than that in the NLF group, but the difference was not statistically significant (*P* = 0.418). The comparisons of MR measurements between NLF and ALF groups are presented in Table 3.

**Table 3:** Comparison of MR measurements between the two groups.

	**NLF group**	**ALF group**	***P***
RLV (mL)	1208.44 ± 235.28	1045.04 ± 210.98	<0.001*
T1-pre (ms)	774.50 ± 76.67	787.28 ± 108.09	0.418
T1-post (ms)	248.78 ± 68.24	330.59 ± 121.30	<0.001*
ΔT1 (ms)	525.71 ± 79.04	456.69 ± 156.04	0.010*
ΔT1% (%)	67.72 ± 7.89	56.93 ± 20.64	<0.001*
Volume-assisted ΔT1 (ms.mL)	636426.53 ± 166461.47	480609.78 ± 209927.78	<0.001*
Volume-assisted ΔT1% (mL)	82139.19 ± 19294.02	59202.27 ± 26221.97	<0.001*

* *The difference is statistically significant*.

### ROC Analysis of all MR Measurements in Predicting Abnormal Liver Function

**Figure 4:**
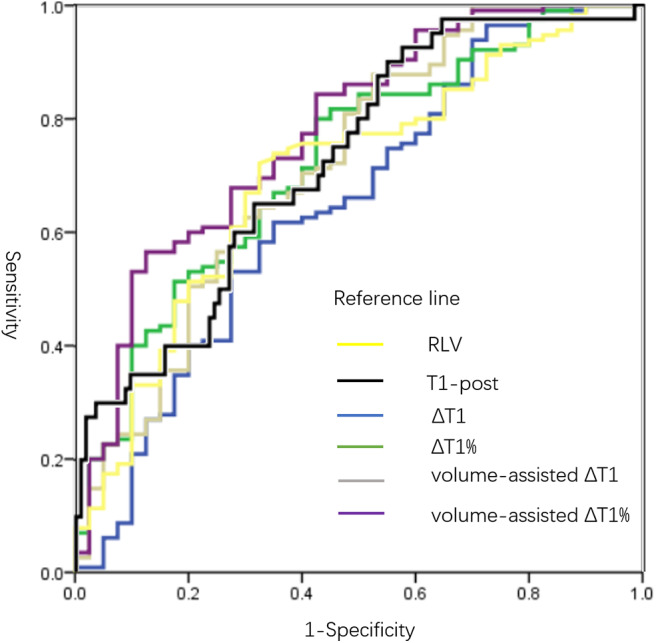
ROC curves of all MR measurements in predicting ALF. AUROCs of RLV, T1-post, ΔT1, ΔT1%, volume-assisted ΔT1 and volume-assisted ΔT1% were 0.702 (95% CI: 0.608–0.796), 0.727(95% CI: 0.640–0.815), 0.647 (95% CI: 0.543–0.752), 0.723 (95% CI: 0.632–0.814), 0.719 (95% CI: 0.621–0.816), and 0.777 (95% CI: 0.691–0.863), respectively. The AUROC of volume-assisted ΔT1% was significantly higher than the AUROCs of the other parameters (*P* < 0.05 for all). T1-pre, T1 relaxation time before Gd-EOB-DTPA injection; T1-post, T1 relaxation time 20 min after Gd-EOB-DTPA injection; RLV, residual liver volume; ΔT1, absolute reduction of T1 relaxation time; ΔT1%, reduction rate of T1 relaxation time; volume-assisted ΔT1, ΔT1*RLV; volume-assisted ΔT1%, ΔT1%*RLV; NLF, normal liver function; ALF, abnormal liver function; ROC, receiver operating characteristic; AUROC, area under the ROC curves.

As revealed by ROC analyses, the AUROCs of RLV, T1-post, ΔT1, ΔT1%, volume-assisted ΔT1 and volume-assisted ΔT1% were 0.702 (95% CI: 0.608–0.796), 0.727(95% CI: 0.640–0.815), 0.647 (95% CI: 0.543–0.752), 0.723 (95% CI: 0.632–0.814), 0.719 (95% CI: 0.621–0.816), and 0.777 (95% CI: 0.691–0.863), respectively. Among them, the AUROC of volume-assisted ΔT1% was significantly higher than the AUROCs of the other parameters (*P* < 0.05 for all). The diagnostic sensitivity, specificity, and AUROCs of all MR measurements are shown in Table 4 and Figure 4.

**Table 4:** Diagnostic sensitivity, specificity, and areas under ROC curves (AUROCs) of MR measurements.

**Parameter**	**AUROC**	**95% CI**	**Cut-off value**	**Sensitivity**	**95% CI**	**Specificity**	**95% CI**
RLV	0.697	0.618–0.768	≤ 1084	67.50%	50.9–81.4%	72.17%	63.0–81.1%
T1-post	0.714	0.636–0.783	>226.81	90.00%	76.3–97.2%	45.22%	35.9–54.8%
ΔT1	0.616	0.535–0.693	≤ 504.40	65.00%	48.3–79.4%	61.74%	52.2–70.6%
ΔT1%	0.701	0.623–0.772	≤ 62.47	57.50%	40.9–73.0%	80.00%	71.5–86.9%
Volume-assisted ΔT1	0.705	0.627–0.776	≤ 4.71*10	47.50%	31.5–63.9%	87.83%	80.4–93.2%
Volume-assisted ΔT1%	0.770	0.696–0.834	≤ 76081.70	87.50%	73.2–95.8%	56.52%	41.0–65.7%

## Discussion

Pretreatment liver function estimation plays a pivotal role in patient evaluation and management (5). Our study showed that Gd-EOB-DTPA-enhanced MRI functional images (T1 mapping parameters) and anatomic information (RLV) were significantly correlated with ICG-15 and demonstrated moderate diagnostic performance in evaluating liver function. Among all MR measurements, ΔT1%^*^RLV, which is a combined parameter of RLV and the reduction rate of T1 relaxation time 20 min after contrast agent injection, yielded the strongest correlation with ICG-15 and highest AUROC in predicting ALF. These results indicated that a combination of anatomic and functional images could help better assessing pretreatment liver function in patients with liver tumors than using either anatomic or functional images alone.

Our study showed negative correlations of ΔT1 and ΔT1% with ICG R-15. In other words, a smaller reduction in the T1 relaxation times before and after contrast agent injection can be indicative of worse pretreatment liver function. Hepatocytes with impaired function show decreased ability to take up Gd-EOB-DTPA, resulting in smaller ΔT1 and ΔT1% values. Our findings were in line with the results of several previous studies, which have confirmed that the T1 mapping on Gd-EOB-DTPA-enhanced MRI can be used to estimate liver function (22, 23). Ding et al. (22) reported that T1-post and ΔT1% could help better evaluate liver necro-inflammatory activity grade and fibrosis stage than apparent diffusion coefficient measurements. In another study, Ding et al. (23) revealed that T1-post and ΔT1% were significantly correlated with MELD scores and demonstrated good diagnostic accuracies in differentiating between good and poor liver functions.

In addition, with ICG R-15 as reference standards, Kamimura et al. (24) and Haimerl et al. (15) reported significant correlations between ICG R-15 and T1 relaxation time index. The T1 mapping sequences of the above two studies were obtained by 3D fat-suppressed T1-weighted VIBE with two different flip angles sequences, while we evaluated T1 relaxation time by the Look-Locker method. According to previous preliminary studies (25, 26), the Look-Locker sequence demonstrated a higher accuracy and repeatability and is relatively less influenced by field strength and scanners, such as B1 inhomogeneity, compared with 3D fat-suppressed T1-weighted VIBE with variable different flip angles sequences. Additionally, we evaluated the performance of T1 parameters in predicting ALF according to the Makuuchi Criteria (18). Using these criteria, the extent of the hepatectomy procedure can be determined according to the preoperative ICG R-15 values, and hepatic resection can be performed with almost zero mortality (27).

We also found a significant negative correlation between RLV and ICG R-15 in this study, indicating that higher RLV is predictive of better liver function. This finding was in accordance with that of previous reports. Several studies (28–30) showed that relative RLV calculated using CT or gadoxetic acid-enhanced MRI could predict postoperative hepatic dysfunction. However, as RLV can evaluate liver function based on the qualitatively defined residual liver parenchyma volume, it fails to consider the function of each hepatocyte. For instance, compared with healthy controls, patients with liver cirrhosis may have larger parenchymal liver volumes, but lower hepatocyte function (31). Thus, RLV may fail to evaluate liver function in such a situation.

On the other hand, although T1 mapping parameters can provide quantitative information regarding hepatocyte function with good repeatability, they have limited capacities in revealing the whole liver structure and the number of hepatocytes with normal function. These limitations might explain the relatively small AUROCs of RLV, T1-post, ΔT1 and ΔT1% in evaluating liver function in our study. Therefore, the assessment of liver function depending on T1 relaxation time or liver volume alone may not be sufficient. To overcome the limitation of a single method in the evaluation of liver function, we combined RLV with T1 mapping measurements. In our study, the volume-assisted T1 parameters ΔT1%^*^RLV demonstrated the strongest correlation with ICG R-15 and the highest AUROC in predicting liver function than the other volume and T1 mapping parameters. Thus far, only a few studies have combined liver volume and T1 signal intensity change before and after Gd-EOB-DTPA administration for liver function evaluation. Haimerl et al. (15) found that the volume-assisted index of T1 relaxation time demonstrated a stronger correlation with liver function than the single reduction rate of T1 relaxation time with Gd-EOB-DTPA-enhanced MR relaxometry. In another study, Yoon et al. (16) reported that posttreatment remnant liver function, predicted using the hepatic extraction fraction multiplied by the RLV on Gd- EOB-DTPA-enhanced MRI was negatively correlated with posttreatment ICG R-15. These studies suggested that the combined application of liver anatomical information and metabolism allows a better assessment of liver function, as with our results. However, signal intensities instead of T1 mapping parameters, were evaluated in Yoon's (16) study. However, signal intensities on T1-weighted images are more sensitive to confounding factors including acquisition parameters, MR scanners, and field strengths (12) and thus less accurate and repeatable than T1 mapping parameters. To overcome this limitation, Yoon et al. (17) recently reported another study with B1 inhomogeneity–corrected volumetric T1 maps of Gd-EOB-DTPA enhanced MRI and found that functional liver volume-to-weight ratio (liver volume/patient's weight)was negatively correlated with the development of hepatic decompensation in compensated cirrhosis, with stronger predictive power than all the single imaging parameters. Their study demonstrated that a combination of liver volume and liver T1 measurements can provide more refined information of liver function. However, diagnostic accuracies were not evaluated in their results and consensus on the best way to combine both quantitative and qualitative parameters haven't been reached yet. To the best of our knowledge, we are the first to multiply T1 mapping parameters and the liver volume measurements on Gd-EOB-DTPA-enhanced MRI to evaluate liver function in patients with liver tumors.

Our results may be beneficial for surgical candidates. In previous clinical practice, an RLV≥25–30% in otherwise healthy livers is usually considered to correlate with good post-resection outcomes (32). However, according to our results, volume-assisted ΔT1%, rather than absolute liver volume, should weigh more in clinical decision making. In other words, if the hepatocyte function is good, as revealed by a relatively high ΔT1% value, patients with low RLV may have an opportunity to be in the surgery list. In contrast, if the hepatocyte function is poor, as revealed by a relatively low ΔT1% value, high RLV would be necessary to guarantee adequate postoperative remnant liver function. Thus, the combined measurement of ΔT1% and RLV on Gd-EOB-DTPA-enhanced MRI allows a more accurate assessment of pretreatment liver function than a single method and should be the preferred approach to guide surgical decision making.

Our study has several limitations. First, the ROIs of T1 mapping images were only placed on one slice, and this may be a source of variation that cannot be well-resolved. However, the whole liver of this slice, excluding the marginal areas, larger vessels, and focal lesions, were covered by the ROIs, which should decrease the probability of sampling error. Second, as a single-center prospective study, no external validation was conducted to test our results. Thus, further studies are warranted to validate and refine our reported findings. Third, the number of included patients, especially the patients with abnormal liver function, was relatively small. According to the treatment strategy in our hospital, liver protecting treatments were be given to patients with liver tumor and/or poor liver function in order to restore a child-Pugh A status before admission. Forth, posttreatment liver function and complications of the included patients were not tracked or well-evaluated in this study; thus, we were not able to measure the capability of T1 mapping and RLV analysis on Gd-EOB-DTPA-enhanced MRI in predicting the risk of postoperative liver failure. As the estimation of the postoperative liver function is vital for preoperative patient management, and further studies are warranted to assess the performance of T1 mapping and RLV on Gd-EOB-DTPA-enhanced MRI in predicting postoperative liver function and patient outcomes.

In conclusion, with ICG R-15 as a reference standard, pretreatment liver function can be quantitatively estimated using the T1 relaxation time and RLV on Gd-ROB-DTPA-enhanced MRI in patients with liver tumors, and a combination of T1 relaxation times and RLV can help better evaluate liver function.

## Data Availability Statement

The datasets generated for this study are available on request to the corresponding author.

## Ethics Statement

The studies involving human participants were reviewed and approved by Biomedical Research Ethics Committee, West China Hospital of Sichuan University. The patients/participants provided their written informed consent to participate in this study. Written informed consent was obtained from the individual(s) for the publication of any potentially identifiable images or data included in this article.

## Author Contributions

BS is the guarantor of integrity of entire study. TD designed this study. TD, LC, CX, ZY, and JC did the MR scan and finished data acquisition. TD and HJ analyzed the data, conducted statistical analysis, and prepare manuscript. HJ, JL, and YW edited and revised the manuscript. BS approved this manuscript finally.

## Conflict of Interest

The authors declare that the research was conducted in the absence of any commercial or financial relationships that could be construed as a potential conflict of interest.

## Abbreviations

ICG: indocyanine green retention rate
T1-pre: T1 relaxation time before Gd-EOB-DTPA injection
T1-post: T1 relaxation time 20 min after Gd-EOB-DTPA injection
RLV: residual liver volume
ΔT1: absolute reduction of T1 relaxation time
ΔT1%: reduction rate of T1 relaxation time
volume-assisted ΔT1: ΔT1^*^RLV
volume-assisted ΔT1%: ΔT1%^*^RLV
NLF: normal liver function
ALF: abnormal liver function
ROC: receiver operating characteristic
AUROC: area under the ROC curves
RFA: radiofrequency ablation
TACE: transarterial chemoembolization
LR: liver resection
RLV/TLV: residual to total liver volume ratio
MELD: model of end-stage liver disease
HBP: hepatobiliary phases
VIBE: volume interpolated breath-hold examination
ALT: alanine aminotransferase
AST: aspartate aminotransferase.
